# A portfolio of climate‐tailored approaches to advance the design of marine protected areas in the Red Sea

**DOI:** 10.1111/gcb.15719

**Published:** 2021-06-18

**Authors:** Laura Gajdzik, Thomas M. DeCarlo, Eva Aylagas, Darren J. Coker, Alison L. Green, John E. Majoris, Vincent F. Saderne, Susana Carvalho, Michael L. Berumen

**Affiliations:** ^1^ Red Sea Research Center King Abdullah University of Science and Technology (KAUST) Thuwal Saudi Arabia; ^2^ Present address: Division of Aquatic Resources Department of Land and Natural Resources State of Hawaiʻi Honolulu HI 96813 USA; ^3^ Present address: College of Natural and Computational Sciences Hawaiʻi Pacific University Honolulu HI 96813 USA

**Keywords:** bleaching, blue carbon, connectivity, coral, mangrove, marine protected area, reef fish, seascape genetics, warming

## Abstract

Intensified coastal development is compromising the health and functioning of marine ecosystems. A key example of this is the Red Sea, a biodiversity hotspot subjected to increasing local human pressures. While some marine‐protected areas (MPAs) were placed to alleviate these stressors, it is unclear whether these MPAs are managed or enforced, thus providing limited protection. Yet, most importantly, MPAs in the Red Sea were not designed using climate considerations, likely diminishing their effectiveness against global stressors. Here, we propose to tailor the design of MPAs in the Red Sea by integrating approaches to enhance climate change mitigation and adaptation. First, including coral bleaching susceptibility could produce a more resilient network of MPAs by safeguarding reefs from different thermal regions that vary in spatiotemporal bleaching responses, reducing the risk that all protected reefs will bleach simultaneously. Second, preserving the basin‐wide genetic connectivity patterns that are assisted by mesoscale eddies could further ensure recovery of sensitive populations and maintain species potential to adapt to environmental changes. Finally, protecting mangrove forests in the northern and southern Red Sea that act as major carbon sinks could help offset greenhouse gas emissions. If implemented with multinational cooperation and concerted effort among stakeholders, our portfolio of climate‐tailored approaches may help build a network of MPAs in the Red Sea that protects more effectively its coastal resources against escalating coastal development and climate instability. Beyond the Red Sea, we anticipate this study to serve as an example of how to improve the utility of tropical MPAs as climate‐informed conservation tools.

## Graphical abstract

A marine protected area (MPA) design for the Red Sea that buffers against the effects of global climate change includes coral reefs that vary in bleaching susceptibility over time: from unbleached/healthy, to heat‐stress‐ and upwelling‐induced bleaching, to recovering‐from‐bleaching reefs. Crucially, such MPA design also accounts for seascape genetics by ensuring these reefs are genetically and physically (through mesoscale eddies) connected to promote recovery and adaptation. Finally, a climate‐informed MPA design helps offset carbon dioxide emissions (CO_2_) by protecting high blue carbon areas, which are large mangrove forests that uptake CO_2_ from the atmosphere via photosynthesis and sequester it in sediments.

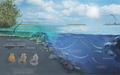

## 1 INTRODUCTION

Marine coastal ecosystems provide goods and services that sustain the livelihood of hundreds of millions of people (Hoegh‐Guldberg, 2011). They act as nurseries and feeding grounds for numerous commercially important species, supporting local fisheries (Cinner et al., 2020). Despite their considerable value, marine ecosystems (e.g., coral reefs, seagrass beds, mangroves) are threatened by anthropogenic stressors (Halpern et al., 2008; Roberts, 2002). Global climate change with its associated rapid warming and recurring extreme climate events has caused the die‐offs of many habitat‐forming species, including corals and seagrasses (Hughes, Anderson, et al., 2018; Hughes, Kerry, et al., 2018; Nowicki et al., 2019). While the pervasive effects of climate change proceed largely unchecked, local anthropogenic activities further jeopardize the health and functioning of marine ecosystems by inducing eutrophication, fragmenting habitats, and over‐harvesting resources (Bellwood et al., 2004; Burkholder et al., 2007; Gray, 1997). These threats primarily arise from the unprecedented coastal development that many nations support to diversify and boost their economy (Neumann et al., 2015).

### 1.1 Intensified local anthropogenic activities due to unprecedented coastal development

**FIGURE 1:**
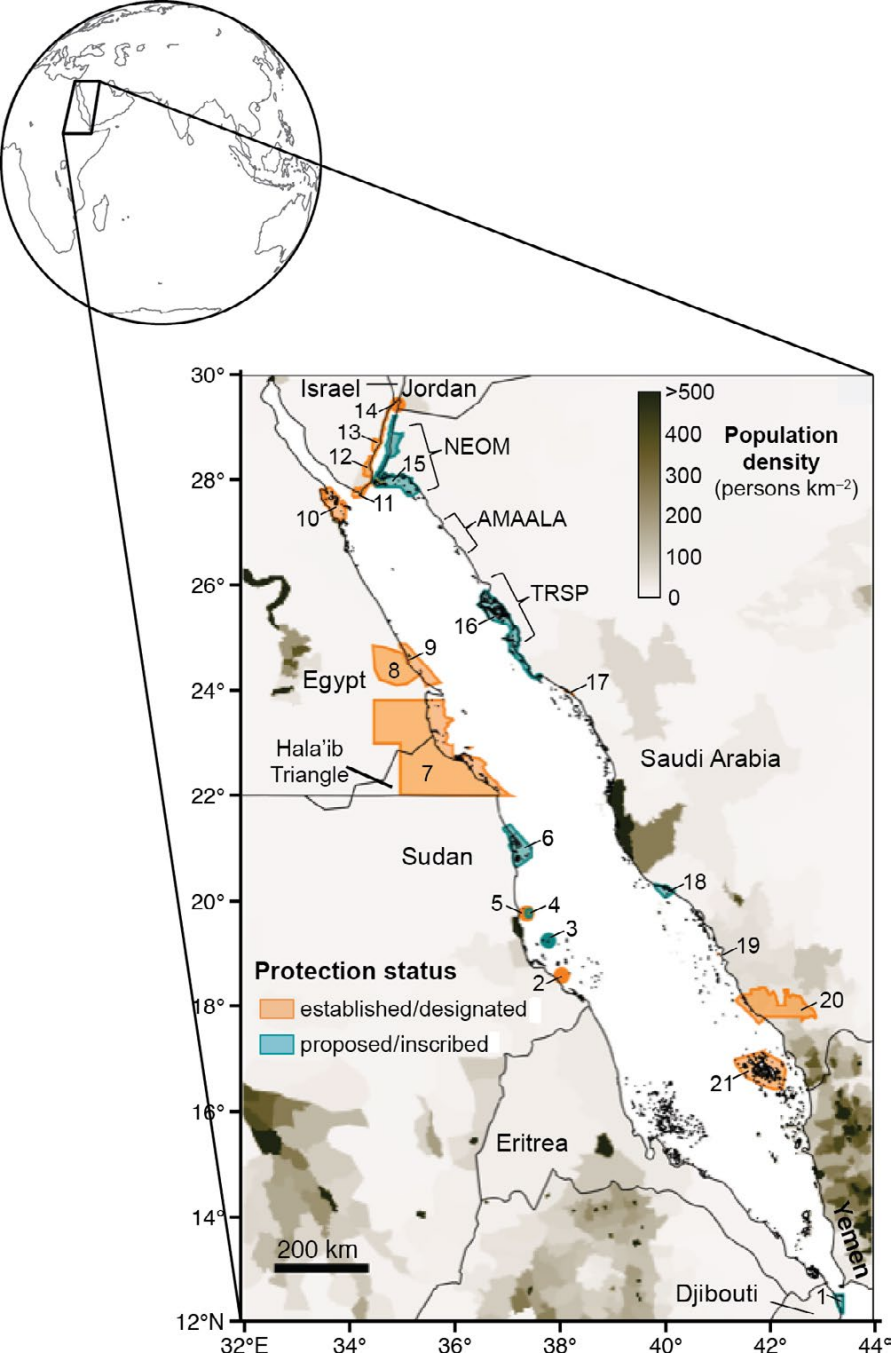
Marine protected areas and human pressures in the Red Sea. The number indicates each individual marine protected area (MPA). The orange‐filled MPAs are either established or designated and are legally recognized, whereas teal‐colored protected areas are proposed or inscribed in the World Heritage Convention but not managed legally or via other means (UNEP‐WCMC & IUCN, 2020). Population density (CIESIN, 2018; persons per km^2^) is shown by a gradient of brown colors and reflects the distribution of the current human pressure along the Red Sea's perimeter. NEOM, AMAALA, and “The Red Sea Project” (TRSP) are three large development projects that are either planned or breaking ground along the coast of Saudi Arabia

The Red Sea is a textbook example of species‐rich marine tropical ecosystems that were historically subjected to low human pressure but are currently under increasing demands due to economic development (e.g., Fine et al., 2019). Located on the western periphery of the Indian Ocean, the Red Sea (Figure 1) is a hotspot for marine biodiversity (Roberts, 2002) that hosts 1166 fish and 360 coral species with 15% and 5% of endemism, respectively (Bogorodsky & Randall, 2019; DiBattista et al., 2016). Its narrow, semi‐enclosed, and deep basin extends ~2000 km north to south and is fringed by extensive coral reefs, mangroves, seagrass meadows, and saltmarshes (Rasul et al., 2015). While parts of the Red Sea are virtually pristine due to their remoteness and/or disputed status (e.g., Hala'ib Triangle at the Egypt‐Sudan border), three large coastal projects are breaking ground along the northeastern coast (Figure 1). The first project is NEOM, a renewable‐energy‐based city with a projected million residents (https://www.neom.com/en‐us/). The second and third projects—AMAALA and The Red Sea Project (TRSP)—are intended to become luxurious tourism destinations in Saudi Arabia, attracting an additional one million people on an annual basis (Daye, 2019). These three projects will be located in areas that currently have a low population density (<100 persons per km^2^), will stretch over ~35% of the entire eastern periphery of the basin, and overlap with some proposed marine protected areas (MPAs; Figure 1). Considering the fast‐paced urbanization of the Red Sea, it is of paramount importance to support these coastal development projects in their aspirations regarding environmental protection and sustainability (e.g., Chalastani et al., 2020) to effectively protect the Red Sea's biodiversity.

### 1.2 Paucity of climate considerations in the existing marine conservation efforts

The Red Sea includes 21 nominal marineprotected areas (MPAs) located within the basin and one near the Strait of Bab‐al‐Mandeb in Djibouti (Figure 1; Gladstone et al., 2003), although five more exist in the Gulf of Aden (UNEP‐WCMC & IUCN, 2020). These MPAs emerged from international (e.g., Sanganeb Marine National Park and Dungonab Bay in Sudan), transnational (e.g., the Regional Organization for the Conservation of the Environment of the Red Sea and Gulf of Aden), and regional initiatives (e.g., Khaled bin Sultan Living Oceans Foundation in Saudi Arabia). However, their protection and enforcement status vary greatly (Figure 1). Sixty‐two percent are designated or established MPAs that are primarily managed by legal means (UNEP‐WCMC & IUCN, 2020), although it is unclear whether they are enforced or not. The other 38% of MPAs are only proposed or inscribed to the World Heritage Convention, and have not been legally established as MPAs (UNEP‐WCMC & IUCN, 2020). Marine conservation efforts in the Red Sea have focused primarily on preserving biodiversity and managing human activities to support the economic growth of nations (Pearson & Shehata, 1998; PERSGA/GEF, 1998). As a result, MPA design in the Red Sea has not yet specifically considered design criteria to address climate change. Identifying and protecting climate refugia and areas with historically variable environmental conditions that may withstand future changes more easily are some key protection measures (McLeod et al., 2012). Including such refugia and variable areas may be particularly relevant to buffer against the impacts of mass coral bleaching events, which are projected to occur on an annual basis in the second half of this century (van Hooidonk et al., 2013).

Mass coral bleaching—when corals across entire reefs or regions simultaneously lose the symbiotic algae, revealing the coral's white skeleton—mainly occurs in response to anomalously high water temperature (Glynn, 1993). Depending on the severity of thermal stress, corals may die, adversely affecting the reef‐associated communities (e.g., Robinson et al., 2019). Reefs in the Red Sea are exposed to strong latitudinal gradients in sea surface temperature (SST) and productivity. Reefs in the north that have not bleached yet are thus often considered as “climate refugia” (Fine et al., 2013). In contrast, reefs in other parts of the basin have suffered from recurring bleaching events due to heat stress or combined heat stress and nutrient excess (DeCarlo et al., 2020, 2021; Monroe et al., 2018; Osman et al., 2018). While MPAs will not prevent mass coral bleaching (e.g., Selig & Bruno, 2010), they can help maintain resilience through the protection of key functional organisms and reduce local stressors (Mcleod et al., 2019). Furthermore, building a network of MPAs that includes reefs from regions with heterogeneous drivers of bleaching will decrease the likelihood of all protected reefs bleaching at once, leaving some possibility for recovery. Such recovery could be further promoted if individual MPAs are well connected (Abelson et al., 2016).

While the need to incorporate connectivity in the design of MPAs in the Red Sea has been mentioned previously (e.g., DeVantier et al., 2000; Gladstone et al., 2003; PERSGA/GEF, 1998), empirical connectivity data have not yet been used for MPA network design in the region. This deficiency has resulted in a non‐cohesive network of small‐sized MPAs that may not effectively protect genetic connectivity patterns linking individual MPAs. Many marine organisms have a bipartite life cycle with demersal adults producing dispersive larvae that can connect distant populations up to hundreds of kilometers apart before settling in appropriate benthic habitats (Shanks, 2009). This “population connectivity”—largely influenced by ocean currents, environmental conditions, and larval behavior (e.g., Nanninga & Manica, 2018; Paris et al. 2007)—is essential to ensure the long‐term persistence of species by maintaining genetic diversity and the potential to adapt to environmental changes (e.g., Carr et al., 2017). Most of the Red Sea's basin is subjected to seasonal mesoscale eddies (Zhan et al., 2016) that can either connect or isolate populations of marine organisms (e.g., Nanninga et al., 2014; Raitsos et al., 2017). Conversely, the most southern section of the basin is not subjected to these mesoscale eddies because the narrow gap between the continental shelves on either side does not allow their formation (Figure 1). Consequently, this southern section of the Red Sea has been often posited to limit gene flow, potentially resulting in local adaptation (Berumen et al., 2019; Roberts et al., 2016). Investigating large‐scale genetic connectivity patterns along the latitudinal range in the Red Sea will provide the basis for designing a better‐connected network of MPAs that promotes resilience and recovery for coral reefs and their associated fauna.

Furthermore, none of the design criteria for the Red Sea's MPAs integrated climate change mitigation measures to reduce greenhouse gas emissions. Coastal vegetated ecosystems, including seagrasses, mangroves, and saltmarshes, are increasingly valued for their capacity to store large stocks of organic carbon for centuries to millennia with high burial rates in their sediments (Duarte et al., 2013; Macreadie et al., 2017; Mcleod et al., 2011; Nellemann et al. 2009). Although they occupy 0.2% of the ocean surface, marine coastal ecosystems contribute to 50% of the carbon buried in marine sediments (Duarte et al., 2013). Hence, they represent important sinks of carbon dioxide (CO_2_) that may partially offset anthropogenic carbon emissions (Macreadie et al., 2017). Blue carbon strategies aiming to preserve and/or restore these coastal ecosystems are thus among the most useful ocean‐based solutions. The Red Sea is an ideal place to integrate such strategies. The basin is mainly bordered by widespread *Avicennia marina* mangrove stands with *Rhizophora mucronata* being sporadically present (Zahran, 2010). Despite relatively low carbon sequestration capacity compared to other mangroves around the world (Almahasheer et al., 2017), mangrove coverage in the Red Sea has increased by 12% over the past four decades (Almahasheer et al., 2016). The basin also hosts ubiquitous, species‐diverse seagrass meadows (El Shaffai et al., 2011). Marine conservation efforts should thus focus on gauging which habitats have the highest potential to act as carbon sinks by assessing carbon burial rates (Howard, Sutton‐Grier, et al., 2017), which can then be prioritized for protection within MPAs (Howard, McLeod, et al., 2017).

Together, these examples illustrate how to advance marine conservation efforts in the Red Sea to ensure greater, long‐term benefits for marine biodiversity. Hence, we propose to integrate climate considerations in the MPA design criteria for the Red Sea by focusing on coral bleaching susceptibility, seascape genetics, and blue carbon. First, we delineate regions of homogeneous spatiotemporal patterns of bleaching and their drivers, the latter reflecting similar thermal and nutrient conditions. Then, we investigate the association between latitudinal population genetic patterns and oceanographic features (e.g., current, mesoscale eddies) to identify the main connectivity pathways and the presence of genetic breaks. Finally, we map the location of mangrove forests and seagrass meadows as well as the carbon sequestration potential of mangroves to identify areas of high blue carbon capacity. MPA candidate areas are then proposed based on these climate‐tailored approaches. Beyond scientific assessments, we also provide practical recommendations for the integration of these approaches in marine conservation to improve the resilience of MPAs in the Red Sea and in other marine tropical environments worldwide.

## 2 CLIMATE‐TAILORED APPROACHES TO BETTER PROTECT THE RED SEA

### 2.1 Coral bleaching susceptibility

**FIGURE 2:**
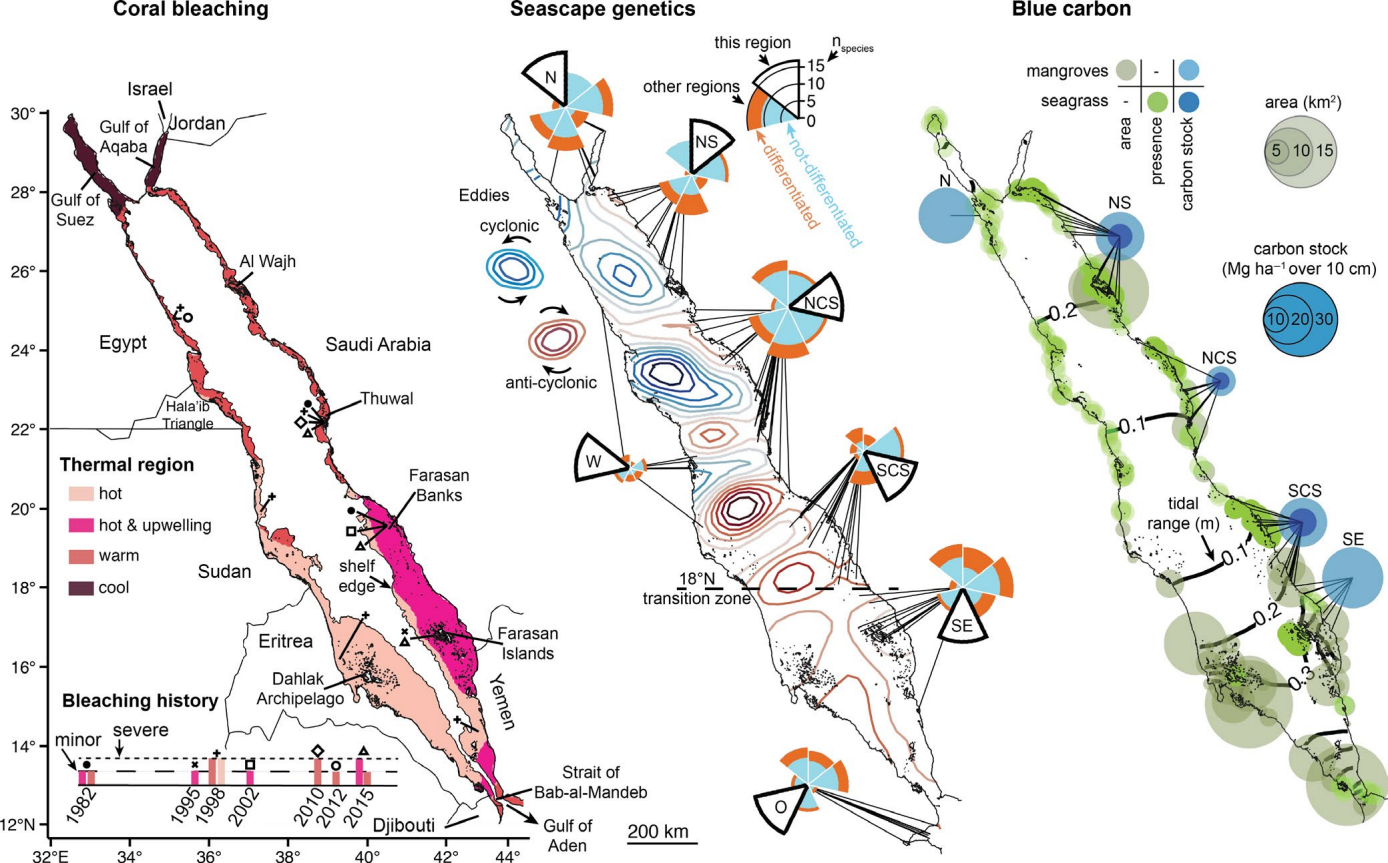
Three climate‐tailored approaches for designing marine conservation efforts in the Red Sea. From left to right: spatiotemporal variability of coral bleaching in relation to thermal regions, seascape genetics reflecting the intimate links between patterns of mesoscale eddies and genetic connectivity, and blue carbon capacity estimated using the stock value of organic carbon in sediments of mangroves (light blue) and seagrasses (dark blue). In the bleaching analysis, four thermal regions were delineated using maximum sea surface temperature (SST): “hot” (light pink) for maxima >31.25℃, “hot and upwelling” for SST >31.25℃ but with summertime dips in temperature due to upwelling (dark pink), “warm” for SST maxima between 28℃ and 31.25℃ (orange), and “cool” (brown) for SST maxima <28℃. The spatial distribution across the basin of recorded minor (large dashed line) and severe (small dashed line) bleaching events is also indicated on the left map with distinct symbols: 1982 (black circle), 1995 (x), 1998 (+), 2002 (white square), 2010 (white diamond), 2012 (white circle), and 2015 (white triangle). Seven regions were identified in relation to seascape genetics: the north (N), northern Saudi (NS), northern‐central Saudi (NCS), southern‐central Saudi (SCS), southeast (SE), west (W), and outside (O) the Red Sea. Each region possesses its own wedge with the proportion of genetic connectivity (turquoise) and genetic differentiation (tangerine orange) between this region and other regions represented by each other wedge moving clockwise starting in the north. Sea surface height anomalies define the cyclonic (gradient of blue colors) and anticyclonic mesoscale eddies (gradient of red colors). The third panel depicts blue carbon in the form of carbon stock values in the sediments of mangroves and seagrass meadows that were expressed in megagram per hectare (Mg ha^−1^) accumulated over 50 years (over 10 cm) within each region. Data are not available for regions O or W. The tide‐influenced distribution and area of mangroves (gradient of moss green colors, km^2^) are illustrated, while only the presence/absence data of seagrass (bright green) are plotted

The Red Sea coastal areas can be divided into four thermal regions reflecting the north‐south water temperature gradient and an east‐west upwelling pattern (Figure 2; additional details about the methodology can be found in Data S1). The highest sea surface temperatures occur in the southern Red Sea, with summertime temperatures occasionally reaching 34℃. Although both sides of the southern Red Sea can be defined as “hot” areas (i.e., climatological maxima >31.25℃), the eastern side is exposed to summertime upwelling, cooling the water by up to 5℃ (DeCarlo et al., 2020). Here, we defined the upwelling region following DeCarlo et al. (2021) as areas that have a minimum daily climatological (1985–2019) mean temperature in July that is less than the maximum daily climatological mean in June. At the northern ends of the Red Sea, the Gulf of Suez and Gulf of Aqaba have the “coolest” surface waters of the Red Sea, with summertime maxima <28℃. Between these northern and southern regions lies a broad “warm” region where summertime maxima range between 28 and 31.25℃.

Crucially, each thermal region has a distinct bleaching history. The southern hot zone experienced widespread coral bleaching in 1998, stretching from Yemen to Eritrea and Sudan (DeVantier et al., 2000; Osman et al., 2018), but no other bleaching events have been reported. Conversely, in the eastern margin of the southern Red Sea where summertime temperatures can be equally high but are modulated by upwelling, the bleaching history is entirely different. Minor bleaching events occurred in 1982, 1995, 1996, and 2002, but severe bleaching and mortality struck in 2015 (DeCarlo et al., 2020; DeVantier et al., 2000; Lozano‐Cortes et al., 2016). These different histories are primarily a result of the complex ways in which upwelling influences bleaching susceptibility. During some years, corals may be spared from bleaching if heat stress is dampened by the cooling effect of upwelling (Riegl & Piller, 2003), whereas corals may experience exacerbated bleaching as a result of upwelled early‐summer nutrients in other years (DeCarlo et al., 2020). In the cool region of the far northern Red Sea, bleaching has yet to be observed (Osman et al., 2018). Experimental evidence has shown that some corals are living up to 4℃ below their bleaching threshold in that region (Krueger et al., 2017), far below the typical 1℃ threshold that applies to most of the world's corals (Baker et al., 2008; Glynn, 1993; Lough et al., 2018; van Hooidonk et al., 2013). Finally, in between the southern and northern extremes, the warm region has experienced repeated bleaching events of varying severity in 1982, 1998, 2010, 2012, and 2015 (DeCarlo et al., 2020; DeVantier et al., 2000; Osman et al., 2018; Figure 2). Additionally, we observed extensive coral bleaching at 25 reef sites to a depth of 30 m (or more) from Thuwal to Al Wajh in October 2020 (A.L.G. and D.J.C pers. obs.; Figure 2). However, reports for this 2020 event are being compiled. Thus, the 1998 bleaching event is currently considered as the most widespread because it spanned the latitudinal range of the Red Sea from Eritrea to Egypt, except the Farasan Banks in the southern upwelling region (DeCarlo et al., 2020; Osman et al., 2018). However, the coral communities in the Farasan Banks were devastated by bleaching and mortality in 2015, whereas the Sudanese reefs (on the opposite side of the basin) were not, despite similar levels of heat stress (DeCarlo et al., 2020). The Red Sea basin is thus delineated into four main thermal regions that differ in spatiotemporal patterns and drivers of bleaching (Figure 2). Of critical importance is that all these thermal regions have not bleached simultaneously in almost three decades of observations.

### 2.2 Seascape genetics

The Red Sea can be roughly divided into four regions based on the succession of seasonal anticyclonic and cyclonic eddies, and variation in surface chlorophyll *a* (Raitsos et al., 2017) that may influence genetic connectivity patterns. Yet, this broad division may not necessarily account for the presence of genetic “barriers” or for patterns of connectivity at specific areas: across the northern gulfs, between eastern and western coastlines, and to the Gulf of Aden.

Here, we used a multi‐taxon genetic approach that integrated pairwise genetic data from 28 species, including fishes (e.g., groupers, damselfishes, goatfishes), corals (e.g., *Pocillopora verrucosa* and *Seriatopora hystrix*), and other benthic organisms (e.g., *Stylissa* sponge, *Heteractis* anemone; Data S1). Our analysis revealed that a stepping‐stone genetic connectivity occurs along two‐thirds of the Red Sea, linking the far north to the central part, and the central part to the western side. No genetic differentiation was observed between the north (N) and northern Saudi (NS) regions, and only small genetic distances between the west (W) and the northern‐central Saudi (NCS) regions (Figure 2). The subdivision in the north highlighted the role of the NS region in genetically connecting the northern sites to the central part of the Red Sea's basin. The NCS region displays the highest degree of genetic similarity with all other regions, especially within the Red Sea (Figure 2), which is in agreement with the formation of more frequent mesoscale eddies in the central area (Raitsos et al., 2013). In contrast, our analysis suggested a higher degree of genetic differentiation in the southern region with greater genetic distances between the southeast (SE) and southern‐central Saudi (SCS) regions than their counterparts in the north. The SE region containing the Farasan Islands is the most genetically differentiated region and exhibits more genetic similarity with regions outside (O) the Red Sea than to those within the basin (Figure 2). This is consistent with oceanographic patterns in that mesoscale eddies do not form adjacent to the Farasan Islands, but rather there is a strong flow into and out of the Red Sea across the Strait of Bab‐al‐Mandeb that connects the Farasan Islands to reef habitats in the Gulf of Aden (Wang et al., 2019). Additionally, the Farasan Islands are characterized by shallow and patchy reefs across the entire shelf, whereas its closest geographic neighbor (i.e., the Farasan Banks) harbors the most diversified reef habitats in the Red Sea (Berumen et al., 2019; Rowlands et al., 2016). These oceanographic and environmental differences make the SE region a transition zone joining the Red Sea to the Gulf of Aden, corroborating a putative genetic barrier at ~18°N (e.g., Nanninga et al., 2014).

The genetic connectivity pathway in the Red Sea is congruent with mesoscale oceanographic patterns in that the distance between neighboring regions, across which few genetic differentiations are found, is similar to the size of eddies. Their velocities can transport water parcels east–west across the basin in 10–20 days (Raitsos et al., 2017). If these seasonal eddies happen to synchronize with spawning events, they could connect populations in adjacent regions and across the basin, even for species that have a relatively short larval duration (e.g., 10 days; Raitsos et al., 2017). Nonetheless, the patterns of genetic connectivity we describe here are species‐ and location‐biased as 80% of the results are based on fish (Data S1) along the eastern margin of the Red Sea. The western side is far less studied (Figure 2), from virtually no genetic information (e.g., the Hala'ib Triangle or the Dahlak Archipelago in Eritrea; Berumen et al., 2019) to a few genetic studies primarily conducted in Sudan (e.g., Priest et al., 2016). As a result, only one region in the western Red Sea has been highlighted and more could be identified in the future. Overall, based on the available data, mesoscale eddies appear to play an important role in maintaining genetic connectivity among regions of the Red Sea.

### 2.3 Blue carbon

Although mangroves and seagrass meadows are latitudinally widespread in the Red Sea, there is relatively limited information about their blue carbon potential. For example, burial rates of organic carbon in sediments were only assessed in the central Red Sea and approximated 15 ± 1 g m^2^ year^−1^ (mean ± standard deviation) and 6.8 ± 1.7 g m^2^ year^−1^ over 10 cm (centimeters) for mangroves and seagrass meadows, respectively (Almahasheer et al., 2017; Serrano et al., 2018). In contrast, various organic carbon stock values are available for both ecosystems, but almost exclusively along the Saudi Arabian coastline (except one site in Egypt; Data S1). These stocks were grouped into the same regions used for seascape genetics and revealed striking latitudinal differences (Figure 2). The greatest carbon stock values are observed for the N and SE regions that approximate the mean global values of buried organic carbon in *Avicennia* stands, 23.3 ± 15.0 Mg ha^−1^(megagrams per hectare) over 10 cm (Atwood et al., 2017). In contrast, the NCS region has the lowest mean carbon stock value of 8.2 Mg ha^−1^ over 10 cm for mangroves (Figure 2). The values of vegetated coastal ecosystems in other nations bordering the Red Sea should also be considered. The Eritrean coastline is indeed covered by large mangrove forests, with some similar in size to those in northern Saudi Arabia, that is, Al Wajh (Figure 2). Additionally, arid saltmarshes might also contribute to the blue carbon of the Red Sea, but little is known about their ecology and distribution. However, based on findings from another Arabian region in the Arabian Gulf (8.1 ± 2.2 Mg ha^−1^ over 10 cm; Cusack et al., 2018), the carbon stock value in sediments of saltmarshes is expected to be lower than those of mangroves.

Overall, the blue carbon capacity of the Red Sea mainly resides in the mangrove forests located in the north and most southern regions, which are also composed by another species of mangroves, *Rhizophora mucronata*. Seagrasses play a more limited role in carbon sequestration in the Red Sea but the central part hosts the largest diversity of seagrass species, that is, 10–12 species out of 60 species recorded worldwide (El Shaffai et al., 2011). Noteworthy, the rates of dissolution of calcium carbonate (CaCO_3_) in the mangroves of the Red Sea could represent another effective mechanism to sink atmospheric CO_2_, up to 20 times more than their carbon burial rates (Saderne et al., 2020).

## 3 BLEACHING‐RECOVERY‐THROUGH‐CONNECTIVITY NEXUS AND HIGH BLUE CARBON SPOTS

Patterns of coral bleaching in the Red Sea vary greatly from region to region and across time (Figure 2). Despite numerous heat‐stress events (Osman et al., 2018), there are no records of significant bleaching in the far north (Krueger et al., 2017), which could be designated as a “bleaching refuge” (Kleinhaus et al., 2020). However, the extent to which the 2020 bleaching event has affected the northern‐central part of the Red Sea is yet to be reported. Preserving this critical area should be a high priority because it will enhance the resilience of the Red Sea to global changes, in a similar way that the Kubulau MPA network (Fiji) was re‐designed to include reefs resilient to bleaching (Weeks & Jupiter, 2013). However, only prioritizing resilient reefs is risky because it relies on the assumption that current patterns of resilience will persist in the future. For example, in the Seychelles, coral bleaching devastated the coral reefs in no‐take zones in 1998 to the extent that fish and benthic communities never fully recovered (Graham et al., 2020). Likewise, the Farasan Banks were considered to be among the most resilient reefs of the Red Sea in 2012 (Rowlands et al., 2012), just 3 years before mass bleaching and mortality in 2015 (DeCarlo et al., 2020). Yet, the destructive bleaching impacts would have occurred regardless of whether the Farasan Banks were protected or not. These examples raise the questions of whether “unbleached” reefs are truly resilient or simply next in line, and whether only preserving currently “healthy” or unbleached reefs remains the best conservation strategy to mitigate against bleaching. Such questioning paves the way for the need to better understand the drivers and patterns of bleaching and how recovery can occur over time. It also resonates with risk‐spreading strategies that focus on preserving habitat heterogeneity and connectivity to favor adaptive evolution.

Expanding protection to include bleached or degraded reefs is part of recent management approaches that propose to assist recovery through networks of MPAs that are better connected (Abelson et al., 2016; Mcleod et al., 2019). The contrasting bleaching histories on either side of the southern Red Sea basin—driven by the exposure to upwelling of cooler waters—offer the potential for eastern and western reefs to serve as “insurance policies” for one another. For example, if a bleaching event kills most of the corals on one side, but reefs on the opposite side are spared, then those remaining reefs could “rescue” the bleached reefs if they are connected through the dispersal of larvae (e.g., via mesoscale eddies: Figure 2). The central region in the Red Sea is a “connectivity hub” that sources larvae to other regions along two‐thirds of the basin (Raitsos et al., 2017). Larvae from the central region may help to replenish depauperate reefs in the Farasan Banks following the 2015 bleaching event. Conversely, in 1998, a majority of reefs in the Red Sea bleached (Figure 2), except the Farasan Banks, which could have provided larvae to other regions at that time. Thus, while the reefs in the central Red Sea should be preserved due to their key role in latitudinal and across‐basin genetic connectivity, protecting some reefs in all thermal regions is essential to maximize the potential for reefs to help one another recover from future bleaching events, wherever they may strike. Similar strategies emphasizing the importance of linking connectivity and climate heterogeneity in MPA design have been proposed for Belize (Mumby et al., 2011) to develop “adaptation networks” that maximize adaptive capacity and phenotypic acclimatization (Webster et al., 2017).

A network of connected MPAs needs not only cover source areas but also “sink” areas to maintain stable populations across connectivity nodes and provide enough larvae that can settle within each MPA (Berumen et al., 2012; Jones et al., 2007). In the Red Sea, the genetic differentiation of the southernmost and hottest region suggests that it hosts different populations of species that could be the most warm‐adapted (Figure 2). Interestingly, this region also appears to be slightly more genetically and oceanographically (Wang et al., 2019) connected to reefs outside the Red Sea (i.e., Djibouti) (Figure 2), where no bleaching was observed in 2015 (Cowburn et al., 2019). This represents the potential for another insurance policy case with replenishment originating from outside the basin boundary. Protecting these reefs might enhance basin‐wide persistence by increasing genetic diversity due to inputs from areas in the Gulf of Aden, which, in turn, may favor future adaptation. Facilitating connectivity might be beneficial for both corals and reef‐associated organisms because healthy coral reefs generally host higher densities of vertebrates and invertebrates (Pratchett et al., 2011), imparting a level of ecosystem resilience (Hock et al., 2017; Mellin et al., 2016). Nevertheless, broad‐scale genetic approaches should be complemented with information at finer spatial scales to refine the size and spacing of MPAs within networks, and to account for movement patterns of adults, juveniles, and larvae of focal species (Green et al., 2015; Weeks et al., 2017).

**FIGURE 3:**
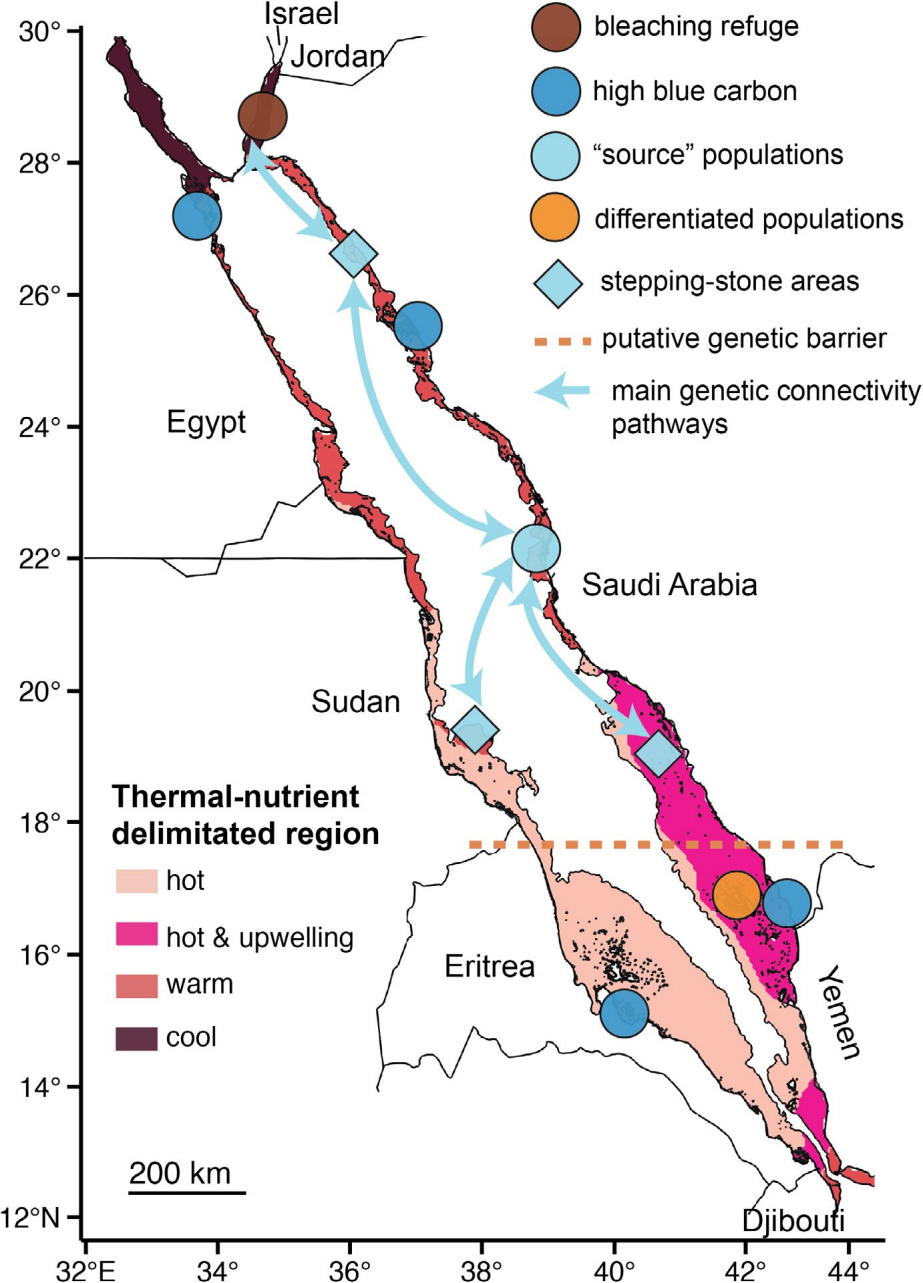
Simplified representation of the three climate‐tailored approaches for the Red Sea showing areas of conservation priority. A climate‐informed MPA network prioritizes (i) bleaching refuge (dark brown circle) and reef areas that vary in bleaching response due to fluctuation in temperatures and nutrient conditions (e.g., upwelling of cold and nutrient‐rich waters) over time that resulted in four main regions (gradient of pink‐brown colors), and (ii) reef areas in each stepping‐stone zone (light blue diamond) that forms the main genetic connectivity pathways (light blue arrow) as well as “source” populations (light blue circle) and genetically differentiated populations (orange circle) that are delimitated by the putative presence of a barrier (dashed orange line). Yet, such MPA network also needs to include areas of high blue carbon potential that act as carbon sinks (dark blue circle) to help offset greenhouse gas emissions

To produce a bleaching‐resilient network of MPAs in the Red Sea and following general conservation guidelines (e.g., Fernandes et al., 2005; Green et al., 2014; McLeod et al., 2009; Salm et al. 2006), we recommend protecting at least three examples of coral communities that sit in each of the four temperature‐nutrient‐delimitated regions (Figure 3). Such a strategy would encompass the whole bleaching spectrum: from healthy, cold‐water reefs in the Gulf of Aqaba that have not yet bleached (“bleaching refuge”) to the repeatedly bleached reefs in central Saudi Arabia, to the hot, unfrequently bleached reefs in Sudan (Figures 2 and 3). Thus, the likelihood that all protected reefs will suffer from bleaching or other local disturbances (e.g., habitat destruction, pollution, and sedimentation) at the same time may be reduced (Green et al., 2014; McLeod et al., 2009).

Yet, to ensure recovery and persistence over time, both reefs in each main stepping‐stone area and “source” reefs in the central Red Sea should be a priority for protection (Figure 3). Our suggestion is to preserve as many replicates and as large areas as possible of these reef communities. The spacing between protected reefs on the same side of the Red Sea basin, and particularly in the southern, genetically‐differentiated region should also not exceed 10–15 km to promote replenishment between individual MPAs as suggested by other studies (D’Aloia et al., 2015; Green et al., 2015; Mora, 2006; Shanks et al., 2003). Such recommendations could also favor basin‐wide acclimatization by, for example, preserving the flexibility in the host–symbiont relationships for corals in the central region (Ziegler et al., 2015).

Better‐connected MPA networks that facilitate bleaching recovery may accommodate climate change more readily, but they do not explicitly mitigate its root cause: greenhouse gas emissions. MPAs can become tools to modulate climate through carbon sequestration (Howard, McLeod, et al., 2017; Roberts et al., 2017). In the Red Sea, mangroves contribute the most to the blue carbon capacity with two hotspots identified (i.e., in Egypt near the Gulf of Suez and in southern Saudi Arabia close to Yemen), and two other potential hotspots (i.e., in Eritrea and northern Saudi Arabia in Al Wajh), which possess widespread mangrove forest area (Figure 3). Protecting blue carbon hotspots areas that are as large as possible should be a conservation priority for the Red Sea. However, areas possessing lower blue carbon capacity should not be entirely neglected. The central Red Sea mangroves and seagrass meadows are a striking example of this because they possess low carbon stock values but have showed some past resilience to anthropogenic disturbances due to sediment accretion rates that tracked closely with those of sea‐level rise since the 20th century (Saderne et al., 2019). In addition to a potential resilience to future sea‐level rise, the capacity of mangroves in the central part to dissolve calcium carbonate and act as carbon sinks may also warrant some level of protection. Additionally, coastal vegetated ecosystems provide important ecosystem services as nursery and feeding grounds for many reef‐associated organisms (Mumby, 2006; Mumby & Hastings, 2007; Saenger et al., 2012) and may assist in maintaining reef health and fisheries (e.g., snappers in the Farasan Banks in the Red Sea or parrotfishes in the Caribbean; McMahon et al., 2011; Mumby & Hastings, 2007). These ecosystems also dissipate wave energy, offering coastal protection against storm surges, erosion, and increasing sea level (Field, 1999; Saderne et al., 2019).

The inclusion of areas with high blue carbon in the MPA network design in the Red Sea would impact the configuration of marine reserves. MPAs in the northern and southern Red Sea**—**particularly in northern Egypt, Eritrea, as well as northern and southern Saudi Arabia where the tallest mangroves were recorded (mean ± SD; 19.8 ± 4.4 m; Shaltout et al., 2020)—should each include large areas of mangrove forests that possess the highest blue carbon capacity. In the central region, replicate reefs adjacent to large perennial seagrass meadows (i.e., *Enhalus acoroides*, *Thallasia hemprichii*, and *Thalassodendron ciliatum*; Qurban et al., 2019) and mangroves should also be included to further increase the blue carbon potential in the Red Sea.

## 4 PRACTICAL RECOMMENDATIONS ON HOW TO DISSEMINATE AND PRODUCE CLIMATE‐TAILORED TROPICAL MPAS

Selecting the location of MPAs and defining their boundaries are fundamental but challenging steps that often require trade‐offs with the available scientific data and rely on the strong communication of clear guidelines to MPA practitioners. Hence, we provide general recommendations on how to incorporate our three proposed climate‐tailored approaches (i.e., coral bleaching susceptibility, seascape genetics, and blue carbon) in the design of tropical MPAs.

A straightforward way to disseminate the scientific information about coral bleaching to researchers and practitioners is to systematically integrate records of bleaching events in a publicly accessible database (Tittensor et al., 2019). Such a database needs to contain high‐resolution spatiotemporal data about bleaching as well as include temperature data derived from remote sensing (e.g., satellites) and *in situ* loggers to map thermal regions. Some examples of bleaching databases are the ReefBase voluntary bleaching database (http://reefbase.org), the expanded global observation bleaching database (Donner et al., 2017a, 2017b), and the Allen Coral Atlas's dynamic brightening monitoring system (Allen Coral Atlas, 2020). In addition to the information contained in these databases, we also suggest to include drivers other than thermal stress such as changes in nutrient regimes, which can further exacerbate bleaching responses. Future trajectories of warming over the next decades and century should also be projected to determine whether the proposed MPAs would be located in warming refugia (i.e., projected stable SST) or in areas that may facilitate adaptation (*sensu* Webster et al., 2017). Following efforts to map coral resilience to thermal stress (e.g., Mumby et al., 2011), mapping the drivers and patterns of coral bleaching over time (such as our Figure 3) as well as including climate projections would guide practitioners in the creation of a bleaching‐resilient MPA network.

Yet, the siting choice of such a MPA network must also align with patterns of genetic connectivity to ensure recovery of reefs that were degraded due to bleaching by maintaining gene flow and favoring *in situ* adaptation. While multi‐taxa genetic studies should be more consistently performed to fully gauge the extent of connectivity patterns, the concordance between such studies and large‐scale oceanographic features in some situations, such as the mesoscale eddies in the Red Sea, indicate that sometimes the main oceanographic pathways may act as surrogates of genetic connectivity of marine organisms if multi‐taxa studies are unavailable. Another compromise may be to use multiple, single‐species genetic studies conducted on foundation (e.g., reef‐building corals or parrotfishes that prevent algae overgrowth of corals) and fisheries species to use connectivity in MPA design to simultaneously maintain ecosystem function, goods, and services for coastal nations. Yet, the effects of global warming on larvae should also be considered as they may develop faster and spend less time in the water column or experience high mortality rates leading to failed recruitment, impacting the level of genetic connectivity between individual MPAs (Munday et al., 2009, 2012). Likewise, climate change will also affect the ocean currents and connectivity by potentially reducing dispersal distances, resulting in the need for MPAs that are geographically closer (Gerber et al., 2014). Once delineated, the oceanographic‐genetic connectivity pathways should be represented in a map that shows genetically‐delimited regions and connectivity hubs and stored in a data layer. Both the map and data layer should be easily accessible and well communicated to practitioners (e.g., Figure 3) so that the genetic information can be translated into effective conservation actions, such as those that we propose for the Red Sea.

Additionally, a bleaching‐resilient, better‐connected MPA network for coral reefs should also be expanded to include vegetated coastal ecosystems with high blue carbon capacity to fight the effects of global warming. Considerable research effort needs to be dedicated to blue carbon science to better quantify the value of the carbon sequestration of marine vegetated ecosystems and their ability to offset CO_2_ emissions (Macreadie et al., 2019). While such efforts continue to expand, a useful rule of thumb may be that stable mangrove forests covering the largest area should be preserved as a first priority, followed by seagrass meadows composed of persistent species (e.g., *Enhalus* spp.), especially if adjacent to other vegetated ecosystems, such as saltmarshes and macroalgae habitats. Additionally, monetizing carbon sequestration through the establishment of a global blue carbon market (Steven et al., 2019) could encourage the uptake of blue carbon initiatives (e.g., voluntary markets) to further help protect coastal vegetated ecosystems (Herr et al., 2015). Hence, researchers should produce a map that displays the locations of the highest blue carbon sinks, which could be then considered during the MPA decision‐making process. This has been attempted by creating the “Mapping Ocean Wealth Explorer,” an online resource where blue carbon values and mangrove restoration potential are illustrated (http://maps.oceanwealth.org/).

## 5 CONCLUSION

The marine policy incentive of this study resides in the creation of a portfolio of climate‐tailored approaches that could boost climate change mitigation and adaptation. One effective way of communicating this portfolio is to produce maps, such as Figure 2, which show (i) thermal regions and bleaching records, (ii) the main genetic connectivity pathway influenced by oceanographic features, and (iii) blue carbon potential. Then, we suggest to provide a simplified version of this portfolio (Figure 3), which is easier to communicate to practitioners and to include in the design narratives. By doing so, this portfolio may result in better‐equipped MPAs facing global warming and guide coastal development projects to reach their sustainability goals.

Yet, such a portfolio needs to consider legal, political, and institutional requirements to be successful (Green et al., 2014). Our analysis revealed that coral reefs in nations bordering the Red Sea share populations of species of fisheries and conservation priority (e.g., Gajdzik et al., 2021; Priest et al., 2016) and crucially serve as insurance policies for one another to mitigate the destructive impacts of coral bleaching. Many of these nations also play a key role in safeguarding blue carbon capacity, which could serve as a ramp to launch the blue economy of the Red Sea (e.g., Cziesielski et al., 2021). This exemplifies that a cohesive MPA network design for the entire Red Sea—as well as the western portion of the Gulf of Aden—needs to be developed following examples of successful multinational partnerships (e.g., Walton et al., 2014) and be managed as a whole. Such a network should be designed using a comprehensive, systematic MPA design process that would consider our climate recommendations along with all other relevant biophysical, social, cultural, and governance considerations (Fernandes et al., 2005; Green et al., 2009, 2014).

Overall, a MPA design integrating the three climate‐tailored approaches will likely offer long‐term resilience to the marine ecosystems in the Red Sea that face greater climate instability and human pressure. Creating such a portfolio for other marine tropical environments represents one avenue to systemically incorporate climate‐mediated data in the core design of marine conservation efforts in tropical regions of the world.

## CONFLICT OF INTEREST

All authors declare no competing interest.

## AUTHORS’ CONTRIBUTIONS

All authors conceived the main ideas and contributed to the collective writing process led by L. Gajdzik. All authors revised the manuscript and gave final approval for publication.

## Supporting information

Supplementary Material

## Data Availability

All data used in this study have been already published and the list of publications has been provided in the supplementary data S1.
