# Supplementary material for: A portfolio of climate‐tailored approaches to advance the design of marine protected areas in the Red Sea
Source: Glob Chang Biol. 2021 Jun 18;27(17):3956–68. doi: 10.1111/gcb.15719 (PMC8453993; doi:10.1111/gcb.15719)
Supplement: Supplementary file 1 — Supplementary Material [file GCB-27-3956-s001.pdf]

Supplementary Materials for

A portfolio of climate-tailored approaches to advance the design of marine protected areas in the Red Sea

Laura Gajdzik*, Thomas M. DeCarlo, Eva Aylagas, Darren J. Coker, Alison Green, John E. Majoris, Vincent F. Saderne, Susana Carvalho, and Michael L. Berumen

*Corresponding author: laura.gajdzik@gmail.com

Data sources

Population density (2020 estimate) was based on the Gridded Population of the World v4.11 (Center for International Earth Science Information Network - CIESIN, 2018) and displayed on Figure 1.

Environmental variables for the Red Sea

Bathymetry data were acquired from Amante and Eakins (2009). We considered the 100-m bathymetric contour to define the shelf edge in the Red Sea on Figure 2. Coastline data were obtained from Wessel and Smith (1996).

Eddies in the Red Sea were visualized using sea surface height anomaly (SSHA) data from the Ssalto/Duacs altimeter products on Figure 2, which were produced and distributed by the Copernicus Marine and Environment Monitoring Service (CMEMS) (marine.copernicus.eu). We also plotted the mean SSHA during 2017.

Tidal range in the Red Sea was based on the M2 tide from the FES2004 model (Lyard, Lefevre, Letellier, & Francis, 2006).

Thermal regions

Sea surface temperature (SST) data were acquired from Coral Reef Watch's CoralTemp1.0 SST product (Liu et al., 2014). We defined "hot" regions as those with maximum monthly mean (MMM) greater than 31.25 °C. Additionally, the upwelling zone is where the climatological minimum daily mean SST in July is less than the climatological maximum daily mean SST in June (Churchill, Bower, McCorkle, & Abualnaja, 2014; DeCarlo et al., 2021). The "cool" zone has MMM <28 °C, and the "warm" zone is all areas with 28 °C < MMM < 31.25 °C.

Coral bleaching history

The history of coral bleaching was compiled from the literature (DeVantier, Turak, Al-Shaikh, 2000; Furby, Bouwmeester, & Berumen, 2013; Monroe et al., 2018; Osman et al., 2018). We considered a bleaching event "severe" if it affected greater than or equal to 30% of corals, and "minor" if an event affected 1-30% of corals. We did not include the 2007 bleaching in Egypt since it was attributed to extreme low tides (causing sub-aerial exposure of corals living on reef flats) and not a thermally induced bleaching event. Additionally, we included minor bleaching events in 1982 that were detected in coral skeletal cores from the NCS region (Fig. 2) and the Farasan Banks in the SCS region (Fig. 2) (DeCarlo et al., 2020).

Genetic connectivity

The genetic connectivity was compiled from 16 studies on genetic connectivity that contained data from at least in two sites in the Red Sea and that presented results of pairwise

genetic distance tables (*i.e.*, fixation indices) with associated significant level. These studies were conducted on 22 fishes including eight butterflyfishes (*Chaetodon melannotus*, *C. austriacus*, *C. fasciatus*, *C. larvatus, C. mesoleucos*, *C. paucifasciatus*, *C. semilarvatus*, and *C. trifascialis*) (DiBattista et al., 2017, 2020), one surgeonfish (*Ctenochaetus striatus*) (DiBattista et al., 2020), one wrasse (*Larabicus quadrilineatus*) (Froukh & Kochzius, 2007), three groupers (*Plectropomus areolatus*, *P. pessuliferus marisrubri*, and *Cephalopholis hemistiktos*) (Priest et al., 2016; Wilson, 2017), one clownfish (*Amphiprion bicinctus*) (Nanninga et al., 2014; Saenz-Agudelo et al., 2015)*,* four damselfishes (*Dascyllus aruanus*, *D. marginatus*, *D. trimaculatus*, and *Chromis viridis*) (Froukh, 2007; Robitzch, 2017; Salas, Bernardi, Berumen, Gaither, & Rocha, 2019)*,* one sea goldie (*Pseudanthias squamipinnis*) (Froukh, 2007), one goatfish (*Mulloidichthys flavolineatus*) (Fernandez-Silva et al., 2015), one snapper (*Lutjanus kasmira*) (DiBattista et al., 2017), and one angelfish (*Pomacanthus maculosus*) (Torquato et al., 2019). We were also able to retrieve genetic distance data from two corals (*Pocillopora verrucosa*, and *Seriatopora hystrix*) (Maier, Tollrian, Rinkevich, & Nürnberger, 2005; Robitzch, Banguera-Hinestroza, Sawall, Al-Sofyani, & Voolstra, 2015), three anemones (*Heteractis magnifica*, *Stichodactyla mertensii*, and *Entacmaea quadricolor*) (Emms et al., 2020), and one sponge (*Stylissa carteri*) (Giles et al., 2015).

The GPS location of all studied sites (used in pairwise comparisons) were plotted using thin black lines and then grouped per region (seven regions in total). If the value of the fixation index between two regions was high and significant, this indicates strong genetic differentiation (i.e., very little genetic exchange). In contrast, if the value of the fixation index was small and not significant, this implied little genetic differentiation (i.e., high genetic exchange). A "significant difference" designation between two regions was only assigned if >50% of sites from one region

were significantly different from >50% of sites from the second region. These results are presented in the pie charts color-coded in Figure 2.

Mangrove coverage, seagrass distribution, and blue carbon

Mangrove areas in the Red Sea were based on data from Almahasheer et al. (2016). The seagrass observations (transformed as presence and absence data) plotted on the map was derived from Bruckner et al. 2012, Jayathilake & Costello, 2018, and Qurban et al., 2019.

The values of the carbon stock sediments (Almahasheer et al., 2016; Arshad, Alrumman, & Eid, 2018; Eid, El-Bebany, & Alrumman, 2016; Eid et al., 2020; Eid & Shaltout, 2016; Garcias-Bonet et al., 2019; Qurban et al., 2019; Serrano, Almahasheer, Duarte, & Irigoien, 2018; Shaltout, Ahmed, Alrumman, Ahmed, & Eid, 2020) (megagram of organic carbon per hectare; $Mg\ ha^{-1}$) were plotted per site and represented by a line on Figure 2. These data have also been standardized over a depth of 10 cm that roughly corresponds to 50 years of accumulation. The carbon stock value in sediments were then averaged and represented per region.

References

Almahasheer, H., Aljowair, A., Duarte, C. M., & Irigoien, X. (2016). Decadal stability of Red Sea mangroves. *Estuarine, Coastal and Shelf Science*, *169*, 164–172. https://doi.org/10.1016/j.ecss.2015.11.027

Amante, C., & Eakins, B. (2009). *ETOPO1 1 Arc-Minute Global Relief Model: Procedures, Data Sources and Analysis. NOAA Technical Memorandum NESDIS NGDC-24*. https://doi.org/doi:10.7289/V5C8276M

Arshad, M., Alrumman, S. A., & Eid, E. M. (2018). Evaluation of carbon sequestration in the sediment of polluted and non-polluted locations of mangroves. *Fundamental and Applied Limnology / Archiv Für Hydrobiologie*, *192*(1), 53–64. https://doi.org/10.1127/fal/2018/1127

Bruckner, A., Rowlands, G., Riegl, B., Purkis, S., Williams, A., Renaud, P. (2012). Khaled bin Sultan Living Oceans Foundation Atlas of Saudi Arabian Red Sea Marine Habitats (p. 262 pp). Panoramic Press.

Center for International Earth Science Information Network - CIESIN. (2018). Gridded population of the world, Version 4 (GPWv4): population Density, Revision 11. https://doi.org/https://doi.org/10.7927/H49C6VHW

Churchill, J. H., Bower, A. S., McCorkle, D. C., & Abualnaja, Y. (2014). The transport of nutrient-rich Indian Ocean water through the Red Sea and into coastal reef systems. *Journal of Marine Research*, *72*(3), 165–181. https://doi.org/10.1357/002224014814901994

DeCarlo, T. M., Carvalho, S., Gajdzik, L., Hardenstine, R. S., Tanabe, L. K., Villalobos, R., & Berumen, M. L. (2021). Patterns, Drivers, and Ecological Implications of Upwelling in Coral Reef Habitats of the Southern Red Sea. *Journal of Geophysical Research: Oceans*, *126*(2). https://doi.org/10.1029/2020JC016493

DeCarlo, T. M., Gajdzik, L., Ellis, J., Coker, D. J., Roberts, M. B., Hammerman, N. M., … Berumen, M. L. (2020). Nutrient-supplying ocean currents modulate coral bleaching susceptibility. *Science Advances*, *6*(34), eabc5493. https://doi.org/10.1126/sciadv.abc5493

DeVantier, L.M., E. Turak, E., K.A. Al-Shaikh, G. D. (2000). Coral communities of the central-northern Saudi Arabian Red Sea. *Fauna of Arabia*, (8), 23–66.

DiBattista, J. D., Gaither, M. R., Hobbs, J.-P. A., Saenz-Agudelo, P., Piatek, M. J., Bowen, B. W., … Berumen, M. L. (2017). Comparative phylogeography of reef fishes from the Gulf of Aden to the Arabian Sea reveals two cryptic lineages. *Coral Reefs*, *36*(2), 625–638. https://doi.org/10.1007/s00338-017-1548-y

DiBattista, J. D., Saenz-Agudelo, P., Piatek, M. J., Cagua, E. F., Bowen, B. W., Choat, J. H., … Berumen, M. L. (2020). Population genomic response to geographic gradients by widespread and endemic fishes of the Arabian Peninsula. *Ecology and Evolution*. https://doi.org/10.1002/ece3.6199

Eid, E. M., El-Bebany, A. F., & Alrumman, S. A. (2016). Distribution of soil organic carbon in the mangrove forests along the southern Saudi Arabian Red Sea coast. *Rendiconti Lincei*, *27*(4), 629–637. https://doi.org/10.1007/s12210-016-0542-6

Eid, E. M., Khedher, K. M., Ayed, H., Arshad, M., Moatamed, A., & Mouldi, A. (2020). Evaluation of carbon stock in the sediment of two mangrove species, Avicennia marina and Rhizophora mucronata, growing in the Farasan Islands, Saudi Arabia. *Oceanologia*, *62*(2), 200–213. https://doi.org/10.1016/j.oceano.2019.12.001

Eid, E. M., & Shaltout, K. H. (2016). Distribution of soil organic carbon in the mangrove *Avicennia marina* (Forssk.) Vierh. along the Egyptian Red Sea Coast. *Regional Studies in*

Marine Science, *3*, 76–82. https://doi.org/10.1016/j.rsma.2015.05.006

Emms, M. A., Saenz-Agudelo, P., Giles, E. C., Gatins, R., Nanninga, G. B., Scott, A., … Berumen, M. L. (2020). Comparative phylogeography of three host sea anemones in the Indo-Pacific. *Journal of Biogeography*, *47*(2), 487–500. https://doi.org/10.1111/jbi.13775

Fernandez-Silva, I., Randall, J. E., Coleman, R. R., DiBattista, J. D., Rocha, L. A., Reimer, J. D., … Bowen, B. W. (2015). Yellow tails in the Red Sea: phylogeography of the Indo-Pacific goatfish *Mulloidichthys flavolineatus* reveals isolation in peripheral provinces and cryptic evolutionary lineages. *Journal of Biogeography*, *42*(12), 2402–2413. https://doi.org/10.1111/jbi.12598

Froukh, T. (2007). *Genetic population structure, gene flow, and evolutionary history of selected ornamental fish in the Red Sea. PhD thesis.* University of Bremen.

Froukh, T., & Kochzius, M. (2007). Genetic population structure of the endemic fourline wrasse (Larabicus quadrilineatus) suggests limited larval dispersal distances in the Red Sea. *Molecular Ecology*, *16*(7), 1359–1367. https://doi.org/10.1111/j.1365-294X.2007.03236.x

Furby, K. A., Bouwmeester, J., & Berumen, M. L. (2013). Susceptibility of central Red Sea corals during a major bleaching event. *Coral Reefs*, *32*(2), 505–513. https://doi.org/10.1007/s00338-012-0998-5

Garcias-Bonet, N., Delgado-Huertas, A., Carrillo-de-Albornoz, P., Anton, A., Almahasheer, H., Marbà, N., … Duarte, C. M. (2019). Carbon and nitrogen concentrations, stocks, and isotopic compositions in Red Sea seagrass and mangrove sediments. *Frontiers in Marine Science*, *6*. https://doi.org/10.3389/fmars.2019.00267

Giles, E. C., Saenz-Agudelo, P., Hussey, N. E., Ravasi, T., & Berumen, M. L. (2015). Exploring seascape genetics and kinship in the reef sponge Stylissa carteri in the Red Sea. *Ecology and Evolution*, *5*(13), 2487–2502. https://doi.org/10.1002/ece3.1511

Jayathilake, D. R. M., & Costello, M. J. (2018). A modelled global distribution of the seagrass biome. *Biological Conservation*, *226*, 120–126. https://doi.org/10.1016/j.biocon.2018.07.009

Liu, G., Heron, S., Eakin, C., Muller-Karger, F., Vega-Rodriguez, M., Guild, L., … Lynds, S. (2014). Reef-scale thermal stress monitoring of coral ecosystems: new 5-km global products from NOAA coral reef watch. *Remote Sensing*, *6*(11), 11579–11606. https://doi.org/10.3390/rs61111579

Lyard, F., Lefevre, F., Letellier, T., & Francis, O. (2006). Modelling the global ocean tides: Modern insights from FES2004. *Ocean Dynamics*, *56*(5–6), 394–415. https://doi.org/10.1007/s10236-006-0086-x

Maier, E., Tollrian, R., Rinkevich, B., & Nürnberger, B. (2005). Isolation by distance in the scleractinian coral *Seriatopora hystri*x from the Red Sea. *Marine Biology*, *147*(5), 1109–1120. https://doi.org/10.1007/s00227-005-0013-6

Monroe, A. A., Ziegler, M., Roik, A., Röthig, T., Hardenstine, R. S., Emms, M. A., … Berumen, M. L. (2018). *In situ* observations of coral bleaching in the central Saudi Arabian Red Sea during the 2015/2016 global coral bleaching event. *PLOS ONE*, *13*(4), e0195814. https://doi.org/10.1371/journal.pone.0195814

Nanninga, G. B., Saenz-Agudelo, P., Manica, A., & Berumen, M. L. (2014). Environmental gradients predict the genetic population structure of a coral reef fish in the Red Sea. *Molecular Ecology*, *23*(3), 591–602. https://doi.org/10.1111/mec.12623

Osman, E. O., Smith, D. J., Ziegler, M., Kürten, B., Conrad, C., El-Haddad, K. M., … Suggett, D. J. (2018). Thermal refugia against coral bleaching throughout the northern Red Sea.

Global Change Biology, *24*(2), e474–e484. https://doi.org/10.1111/gcb.13895
Priest, M. A., DiBattista, J. D., McIlwain, J. L., Taylor, B. M., Hussey, N. E., & Berumen, M. L. (2016). A bridge too far: dispersal barriers and cryptic speciation in an Arabian Peninsula grouper (*Cephalopholis hemistiktos*). *Journal of Biogeography*, *43*(4), 820–832. https://doi.org/10.1111/jbi.12681
Qurban, M. A. B., Karuppasamy, M., Krishnakumar, P. K., Garcias-Bonet, N., & Duarte, C. M. (2019). Seagrass Distribution, composition and abundance along the Saudi Arabian coast of Red Sea. In R. M. A. Najeeb & C. F. Stewart (Eds.), *Oceanographic and Biological Aspects of the Red Sea* (pp. 367–385). Springer Oceanography. Retrieved from http://link.springer.com/10.1007/978-3-319-99417-8_20
Robitzch, V., Banguera-Hinestroza, E., Sawall, Y., Al-Sofyani, A., & Voolstra, C. R. (2015). Absence of genetic differentiation in the coral *Pocillopora verrucosa* along environmental gradients of the Saudi Arabian Red Sea. *Frontiers in Marine Science*, *2*. https://doi.org/10.3389/fmars.2015.00005
Robitzch, V. S. N. (2017). *The Assessment of current biogeographic patterns of coral reef fishes in the Red Sea by incorporating their evolutionary and ecological background. PhD thesis.* King Abdullah University of Science and Technology.
Saenz-Agudelo, P., Dibattista, J. D., Piatek, M. J., Gaither, M. R., Harrison, H. B., Nanninga, G. B., & Berumen, M. L. (2015). Seascape genetics along environmental gradients in the Arabian Peninsula: insights from ddRAD sequencing of anemonefishes. *Molecular Ecology*, *24*(24), 6241–6255. https://doi.org/10.1111/mec.13471
Salas, E. M., Bernardi, G., Berumen, M. L., Gaither, M. R., & Rocha, L. A. (2019). RADseq analyses reveal concordant Indian Ocean biogeographic and phylogeographic boundaries in the reef fish *Dascyllus trimaculatus*. *Royal Society Open Science*, *6*(5), 172413. https://doi.org/10.1098/rsos.172413
Serrano, O., Almahasheer, H., Duarte, C. M., & Irigoien, X. (2018). Carbon stocks and accumulation rates in Red Sea seagrass meadows. *Scientific Reports*, *8*(1), 15037. https://doi.org/10.1038/s41598-018-33182-8
Shaltout, K. H., Ahmed, M. T., Alrumman, S. A., Ahmed, D. A., & Eid, E. M. (2020). Evaluation of the carbon sequestration capacity of arid mangroves along nutrient availability and salinity gradients along the Red Sea coastline of Saudi Arabia. *Oceanologia*, *62*(1), 56–69. https://doi.org/10.1016/j.oceano.2019.08.002
Torquato, F., Range, P., Ben-Hamadou, R., Sigsgaard, E. E., Thomsen, P. F., Riera, R., … Møller, P. R. (2019). Consequences of marine barriers for genetic diversity of the coral-specialist yellowbar angelfish from the Northwestern Indian Ocean. *Ecology and Evolution*, *9*(19), 11215–11226. https://doi.org/10.1002/ece3.5622
Wessel, P., & Smith, W. H. F. (1996). A global, self-consistent, hierarchical, high-resolution shoreline database. *Journal of Geophysical Research: Solid Earth*, *101*(B4), 8741–8743. https://doi.org/10.1029/96jb00104
Wilson, S. (2017). *Assessment of genetic connectivity between Sudan and Saudi Arabia for commercially important fish species. MSc thesis.* King Abdullah University of Science and Technology, Saudi Arabia.
